# Extracellular Vesicles Derived From Lipopolysaccharide‐Challenged Gingival Fibroblast Reveal Distinct miRNA Expression Patterns Associated With Reduced Cancer Survival

**DOI:** 10.1002/cre2.70099

**Published:** 2025-02-18

**Authors:** Daniel Diehl, Charlotte Lauren Brauer, Hagen S. Bachmann, Daniel Pembaur, Patrick Philipp Weil, Anton Friedmann

**Affiliations:** ^1^ Department of Periodontology, School of Dentistry, Faculty of Health Witten/Herdecke University Witten Germany; ^2^ Center for Biomedical Education and Research (ZBAF), Institute of Pharmacology and Toxicology, Faculty of Health Witten/Herdecke University Witten Germany; ^3^ Center for Biomedical Education and Research (ZBAF), Institute of Biochemistry and Molecular Medicine, Faculty of Health Witten/Herdecke University Witten Germany; ^4^ Centre for Biomedical Education and Research (ZBAF), Institute for Clinical Molecular Genetics and Epigenetics, Faculty of Health Witten/Herdecke University Witten Germany

## Abstract

**Objectives:**

Periodontitis is a prevalent inflammatory disease with established systemic implications. Extracellular vesicles (EVs) have emerged as key mediators of intercellular communication, potentially linking periodontitis to systemic diseases. However, the molecular cargo of EVs from inflamed periodontal cells remains poorly characterized. This study investigates the EV cargo of human gingival fibroblasts (hGF‐hTERT) following lipopolysaccharide (LPS) stimulation and explores their potential role in cancer progression.

**Materials and Methods:**

EVs were isolated from LPS‐treated and untreated fibroblasts via ultracentrifugation. Dynamic light scattering and scanning electron microscopy characterized EV size and morphology. RNA sequencing identified differentially expressed miRNAs, validated by qPCR. Functional pathway enrichment and in‐silico analyses using The Cancer Genome Atlas (TCGA) were performed to assess EV‐associated miRNA impact on tumorigenesis.

**Results:**

EV size and concentration remained unchanged after LPS stimulation. However, LPS‐derived EVs exhibited a 2.6‐fold increase in miRNA content, with three significantly upregulated miRNAs: miR‐146a‐5p, miR‐486‐5p, and miR‐451a. Functional enrichment analysis revealed their involvement in inflammation, immune modulation, and cancer pathways. In vitro, LPS‐derived EVs significantly enhanced prostate cancer (LnCap) cell proliferation. TCGA analysis linked the upregulated miRNAs to poor cancer prognosis.

**Conclusions:**

LPS stimulation alters the miRNA cargo of gingival fibroblast‐derived EVs, enhancing pathways associated with inflammation and cancer progression. These findings suggest a mechanistic role for periodontal EVs in systemic disease pathogenesis, warranting further investigation into their diagnostic and therapeutic potential.

## 1 Introduction

According to the 2016 Global Burden of Disease study, periodontitis remains one of the most prevalent conditions in the world, and the evidence for a close association between periodontitis and systemic diseases has been well established (Nazir et al. 2020). In the past three decades, observational and experimental studies have precisely elucidated the systemic risk factors for periodontitis and vice versa. This has led to a profound understanding of periodontal health's impact on general health (Martínez‐García and Hernández‐Lemus 2021). While the clinical evidence for this association has been sufficient to shape contemporary clinical practice in periodontology, our understanding of the molecular and biological constituents that connect periodontitis with other diseases—whether inflammatory, neurological, or oncological—has remained relatively superficial, on the other hand (Cullinan and Seymour 2013). Currently, evidence for the systemic pathophysiologic consequence of periodontitis is limited to reports about elevated blood levels of inflammatory cytokines and chronic exposure to periodontal pathogens (Cullinan and Seymour 2013; Winning and Linden 2017). However, the idea that the root of these related diseases is due to the systemic inflammatory burden in periodontitis patients is widely discussed. Depending on the study design and possible confounding factors, different clinical studies have revealed heterogeneous results regarding serum cytokine levels in periodontitis patients (Andrukhov et al. 2011; Cetinkaya et al. 2013). Therefore, further studies into putative mechanisms underlying the influence of periodontitis are warranted.

Extracellular vesicles (EVs) are a heterogeneous group of cell‐derived nanoparticles ranging from 30 to 1000 nm in size. They comprise a lipid bilayer that encloses a subset of proteins, lipids, and nucleic acids from the parent cell (Robbins and Morelli 2014). Initially considered waste disposal organelles, EVs have increasingly been shown to have a central role in mediating physiological and pathological processes (Abels and Breakefield 2016). EVs are either blebbed off the cell membrane (microvesicles) or produced within multivesicular bodies (exosomes) and then released into the extracellular space through exocytosis. They interact with other cells by binding to receptor proteins, releasing their contents into the cell, or being taken up by endocytosis. Once inside the cell, the metabolites, proteins, and nucleic acids (such as mRNA, lncRNA, or miRNA) may trigger or regulate various signaling pathways. The protein cargo of EVs is highly conserved and is consistent across EVs of different tissues. Therefore, mRNA and noncoding RNA are recognized as the primary mediators of EV‐mediated communication between cells. Because EV cargo has been shown to reflect the state of the originating cell, they have drawn increasing attention from researchers of all fields as putative biomarkers and regulators of various pathologies. Furthermore, their lipid envelope allows them to penetrate multiple biomembranes, such as the blood–brain barrier, while carrying and preserving even the most fragile RNA species.

Consequently, these features are particularly interesting to further our understanding of the molecular mechanism underlying the connection between periodontitis and systemic diseases. Extensive research has revealed a significant pathomechanistic effect of exosomes derived from chronically inflamed tissues. For instance, activated monocyte‐derived exosomes constitute a substantial contributor to the development of atherosclerosis. Various in vitro studies reported that inflammatory exosomes may activate pro‐inflammatory transcription factors, promoting vascular endothelial cell activation and, thus, atherosclerotic plaque formation (Gao et al. 2016; Tang et al. 2016). Moreover, circulating exosomes have been shown to worsen stroke outcomes in a murine model (Zhang et al. 2021). These findings are underlined by a series of studies investigating serum exosomes from patients suffering from systemic lupus erythematosus (SLE) or Crohn's disease, where a significant immunomodulatory effect of the vesicles, mediated by regulatory microRNAs, has been reported (Gong et al. 2022; Lee et al. 2016).

However, studies investigating the cargo and function of periodontal cell EVs are lacking. Therefore, this in vitro proof of principle study aimed to characterize the EV cargo and release of periodontal fibroblasts challenged with lipopolysaccharide (LPS) compared to fibroblasts in a physiologic state. Moreover, we investigated how EVs from inflamed periodontal cells may contribute to the development and progression of cancer.

## 2 Materials and Methods

### 2.1 Cell Culture

Human telomerase‐immortalized gingival fibroblasts (hGF‐hTERT; Applied Biological Materials Inc., Richmond, Canada) were grown in Dulbecco's modified Eagle's medium (PAN‐Biotech, Aidenbach, Germany) supplemented with 10% (v/v) fetal bovine serum (FBS; PAN Biotech), 100 U/mL penicillin, and 100 μg/mL streptomycin (PAN‐Biotech). These TERT‐immortalized cells share the benefits of primary cells along with those of immortalized cell lines, ensuring a stable phenotype over a high passage number (Lee et al. 2004). For EV purification, the cells were seeded into 150 mm culture dishes at a density of 4 × 10^6^ cells per dish and incubated with 35 mL of either LPS‐supplemented (10 μg/mL) or LPS‐free culture medium for 96 h. To validate the expression of miRNA under these conditions, hGF‐hTERT were seeded into 6‐well culture plates at a density of 3 × 10^5^ and incubated with the same media for 96 h. To evaluate the isolated effect of EV on other cells, the prostate cancer cell line LnCap, cultured in Roswell Park Memorial Institute medium (PAN‐Biotech) supplemented with 20% FBS and the same antibiotic was seeded into six‐well culture plates at a density of 3 × 10^5^ and incubated with 20 particles per cell (ppc) of either LPS‐ or control‐derived EVs dissolved in 1 mL of fresh culture medium per well for 48 h.

### 2.2 Purification of EVs

EVs were isolated from culture media by differential ultracentrifugal separation. In brief, harvested supernatants were centrifuged at 2800 × *g* for 20 min to pellet cell debris, followed by ultracentrifugation (100,000 × *g*, 90 min, 4°C) on an Optima L‐90K (Beckmann Coulter, Brea, CA, USA) using the SW28 swing‐out bucket rotor and 38.5 mL Open‐Top, thin‐walled polypropylene tubes (Beckmann Coulter). The EV pellets were washed in 2 mL ice‐cold PBS and subjected to further purification on a Sorvall RC M120 GX (150,000 × *g*, 90 min, 4°C, Thermo Fisher Scientific Inc., Waltham, WA, USA) using an S55‐S swinging bucket rotor (Thermo Fisher Scientific Inc.). Final EV pellets were dissolved in 150 μL PBS and immediately stored at −80°C.

### 2.3 Physical Characterization of EVs by Dynamic Light Scattering (DLS)

DLS was used to determine the hydrodynamic diameter and the concentration of the EVs. For DLS measurement, 100 µL of EV‐suspension was diluted with 900 µL of DPBS (PAN‐Biotech) and transferred to a glass cuvette with a square aperture. The suspension was subjected to a particle concentration measurement in a Zetasizer Ultra Red (Malvern Panalytical, Malvern, GB). Each measurement was repeated five times, and all samples were analyzed with identical settings (material: protein, dispersant: water, temperature: 25°C, equilibration time: 120 s). Data quality was assessed, and samples with large aggregates were excluded from further experiments. To determine EV concentration, particle peak concentrations of particles with sizes between 90 and 530 nm were summed.

### 2.4 Scanning Electron Microscopy (SEM)

SEM was used to analyze the morphology and structure of purified EVs. Samples were fixated using glutaraldehyde on an 8 × 8 mm tissue sponge and secondary fixation was done using osmium tetroxide. After dehydrating and drying the samples with the Critical Point Dryer (Bal‐Tec AG, Balzers, Liechtenstein), the tissue sponges containing the EV samples were mounted on studs and coated with gold/palladium and finally sputter coated with the Sputter SCD 050 (Bal‐Tec AG, Balzers, Liechtenstein)—both according to manufacturer's instructions. After sample preparation was completed, they were visualized under the scanning electron microscope (Carl Zeiss AG, Oberkochen, Germany).

### 2.5 RNA Extraction and Quality

RNA extraction from cells and purified EVs was executed using the TRIzol method (Simms et al. 1993). The RNA concentration and purity were measured using ultraviolet‐visible spectrophotometry (NanoPhotometer P‐Class, Implen GmbH, Munich, Germany) via the optical density (OD) at 260 and 280 nm. An OD_260/280_ of 1.9–2.1 was classified as protein‐free RNA. The integrity (RIN) and quality of the total isolated RNA were measured on an Agilent 2100 Bioanalyzer (Agilent Technologies Inc., Waldbronn, Germany) according to the manufacturer's instructions.

### 2.6 MicroRNA First Strand and Complementary DNA (cDNA) Synthesis

cDNA was synthesized using the Mir‐X miRNA First‐Strand Synthesis kit for miRNA or the PrimeScript RT Master Mix for mRNA (Takara Bio, Kusatsu, Japan). Briefly, 1 μg of RNA was reverse‐transcribed following the manufacturer's instructions. The thermocycler T100 (Bio‐Rad Laboratories, Hercules, California) was programmed for a reverse transcription step of either 60 or 15 min at 37°C, respectively, followed by a 5‐s inactivation step at 85°C and a holding temperature of 4°C. The cDNA samples were immediately stored at −20°C.

### 2.7 Library Preparation and RNA Sequencing

Library preparation was conducted according to the manufacturer's instructions using the Single Indexing Library Preparation Kit (Illumina, San Diego, CA, USA) and miND spike‐ins (TAmiRNA, Vienna, Austria) for quality control and data normalization. Next, the libraries were run through a high‐pass filter for size purification enhancement of small RNA yield (Blue Pippin, Sage Science Inc., Beverly, MA, USA). Afterward, the library quality control was completed using the Bioanalyzer DNA 1000 chip (Agilent 2100 Bioanalyzer System, Agilent Technologies). Lastly, the samples were sequenced using the Illumina Hi‐Seq. 2500 sequencing machine (Illumina, San Diego, CA, USA) with approximately 10 million reads per sample.

### 2.8 Quantitative Real‐Time Polymerase Chain Reaction

For the quantitative polymerase chain reaction (qPCR) amplification, we utilized a CFX96 Touch Real‐Time PCR Cycler. In each well, we added 20 μL of master mix, which comprised 10 μL of 2X iTaq Universal SYBR Green Supermix, 1 μL of the respective cDNA solution, 0.3 μL of each primer pair (10 pmol per primer), and 8.4 μL of nuclease‐free H2O (Fresenius Kabi, Bad Homburg, Germany). The amplification process involved 40 cycles, starting with an initial denaturation at 95°C for 3 min, then denaturation at 95°C for 10 s, annealing at the respective Ta for 30 s, and extension at 70°C for 10 s. All PCR primers were designed to include an exon–exon junction with amplicon lengths ranging from 70 to 200 nt nucleotides (Table 1).

**Table 1:** Primer sequences for RT‐qPCR.

Gene	Accession number	5′→3′ Forward primer	3′→5′ Reverse primer
miR‐146a‐5p	MI0000477 (miRBase)	TGAGAACTGAATTCCATGGGTT	Takara Bio Cat.# 638316
miR‐451a	NC_000017.11	AAACCGTTACCATTACTGAGTT	Takara Bio Cat.# 638316
miR‐486‐2‐5p	NC_000008.11	TCCTGTACTGAGCTGCCCCGAG	Takara Bio Cat.# 638316
U6	NC_000015.10	Takara Bio Cat.# 638316	Takara Bio Cat.# 638316
VIM	NM_003380.5	GCCCTTGACATTGAGATTGCCA	TCAACCAGAGGGAGTGAATCCA
CDH1	NM_001308176.2	AAGAACGCCAGGCCAAACAA	TGCAGCTGGCTCAAGTCATA
SEC. 63	NM_007214.5	GAGATCAGAATGCCGAGCAA	CCCATCCTGCAAGCAGAACTA

For miRNA amplification, we used the entire sequence of the respective mature miRNA (21–23 nt) as the miRNA‐specific 5′‐primer, while the mRQ 3′‐primer provided with the First Strand synthesis kit (Takara Bio) was employed as the 3′‐primer for qPCR. Amplification was conducted following the previously mentioned protocol, except that an annealing step was performed for 20 s at 60°C, while the extension step was omitted due to the short amplicon length (21–23 nt). The genes *U6* (U6 spliceosomal RNA) and *SEC. 63* were used as internal reference genes.

### 2.9 Proliferation Assay

LnCap cells were seeded to 96‐well culture plates at a density of 2 × 10^4^ cells per well and left to adhere overnight. The next day, culture media were supplemented with respective EV preparations at a concentration of 20 ppc and cultivated for another 48 h. Finally, cells were incubated with a resazurin‐based fluorescent viability stain (Presto Blue HS, Thermo Fisher Scientific Inc.) for 2 h. After incubation, the fluorescence of viable cells was measured at an excitation of 560 nm and an emission of 590 nm using a multiplate reader (Tecan). All treatments were run in triplicate on each plate of three distinct biological experiments.

### 2.10 Data Processing

The overall quality of the NGS data was evaluated with fastQC v0.11.9 (https://www.bioinformatics.babraham.ac.uk/projects/fastqc/) and multiQC v1.10 (Ewels et al. 2016). Reads from all passing samples were adapter trimmed and quality filtered using cutadapt v3.3 (https://cutadapt.readthedocs.io/en/stable/) and filtered for a minimum length of 17 nt. Mapping steps were performed with bowtie v1.3.0 (Langmead et al. 2009) and miRDeep2 v2.0.1.2 (Friedländer et al. 2012). Reads were mapped first against the genomic reference GRCh38.p12 provided by Ensembl (Zerbino et al. 2018) allowing for two mismatches. Subsequently, miRBase v22.1 (Griffiths‐Jones 2004) was used to filter for miRNAs of *homo sapiens* only, allowing for one mismatch. For a general RNA composition overview, non‐miRNA mapped reads were mapped against RNAcentral (2019) and then assigned to various RNA species of interest. Statistical analysis of preprocessed NGS data was done with R v4.0 and the packages pheatmap vNA, pcaMethods v1.82 and genefilter v1.72. Differential expression analysis with edgeR v3.32 (Robinson et al. 2010) used the quasi‐likelihood negative binomial generalized log‐linear model functions provided by the package. The independent filtering method of DESeq. 2 (Love et al. 2014) was adapted for use with edgeR to remove low abundance miRNAs and thus optimize the false discovery rate correction.

### 2.11 Target Gene and Pathway Enrichment Analyses

Target gene predictions were performed for miRNAs exhibiting a log2(fold change) > 1.5 with a −log(*p*) > 2 over the control EVs, resulting in three overexpressed miRNAs. Computational target prediction was performed with TargetScan (Agarwal et al. 2015) and mirTarBase (Huang et al. 2022). Only experimentally verified targets exhibiting an adjusted *p* ≤ 0.05 were included in miRNA‐centric network visual analytics using miRNet 2.0 (https://www.mirnet.ca) (Chang et al. 2020). For functional annotation of thusly identified target genes, Kyoto Encyclopedia of Genes and Genomes (KEGG) pathway enrichment analysis was performed using the online Database for Annotation, Visualization, and Integrated Discovery (DAVID v202, www.david.ncifcrf.gov) (Huang et al. 2009).

### 2.12 In‐silico Analyses on Clinical Cancer Expression Data

The Cancer Genome Atlas (TCGA) repository at the Genomic Data Commons (GDC) Data Portal was queried for miRNA expression analysis in all associated cancers according to KEGG via UCSC Xena (http://xena.ucsc.edu) (Goldman et al. 2020). miRNA expression data was collected from lung small cell carcinoma (LUSC), prostate carcinoma (PRAD), pancreatic adenocarcinoma (PAAD), colorectal adenocarcinoma (COAD), breast cancer (BRCA), and head and neck squamous carcinoma (HNSC). The relative fold changes of EV‐related miRNA expression between the Tumor and Normal adjacent tissues were calculated by Student's *t*‐test based on the log2(RPM + 1) values provided by TCGA. For all individual cancer data sets, *Z*‐scores were calculated in relation to mean miRNA expression in adjacent healthy tissue samples. Subsequently, a multivariate Cox proportional hazards regression was performed for all covariates, including age, histologic grade, and the expression levels (as *z*‐scores) of selected three miRNAs. Additionally, estimated survival curves were calculated applying the weighted parameter coefficients from the Cox regression of EV‐associated miRNA to investigate a hypothetical influence on cancer survival. All statistical analyses were performed in GraphPad Prism 9, and *p* ≤ 0.05 were considered significant.

### 2.13 Statistical Analysis

RT‐qPCR data were analyzed using the $2^{‐∆∆C_{t}}$ method (Schmittgen and Livak 2008). In brief, Δ*C*_t_ values were obtained by subtracting target gene values (Ct_GOI_) from the reference gene (Ct_REF_). Next, ΔΔ*C*_t_ was calculated utilizing Student's *t*‐test. Finally, mean differences and SEM were transformed ($2^{‐∆∆C_{t}}$) to obtain the relative fold change. For the proliferation assay, data were normalized to the control (100%), and mean and standard deviation were calculated for each group. A significant increase in viable cells was determined by analysis of variance, followed by Dunnett's multiple comparisons tests. All experiments were conducted with three distinct biological replicates. Differences with *p* < 0.05 were considered significant.

## 3 Results

### 3.1 Gingival Fibroblast‐Derived EVs Exhibit Differential Cargo After LPS Challenge

**Figure 1:**
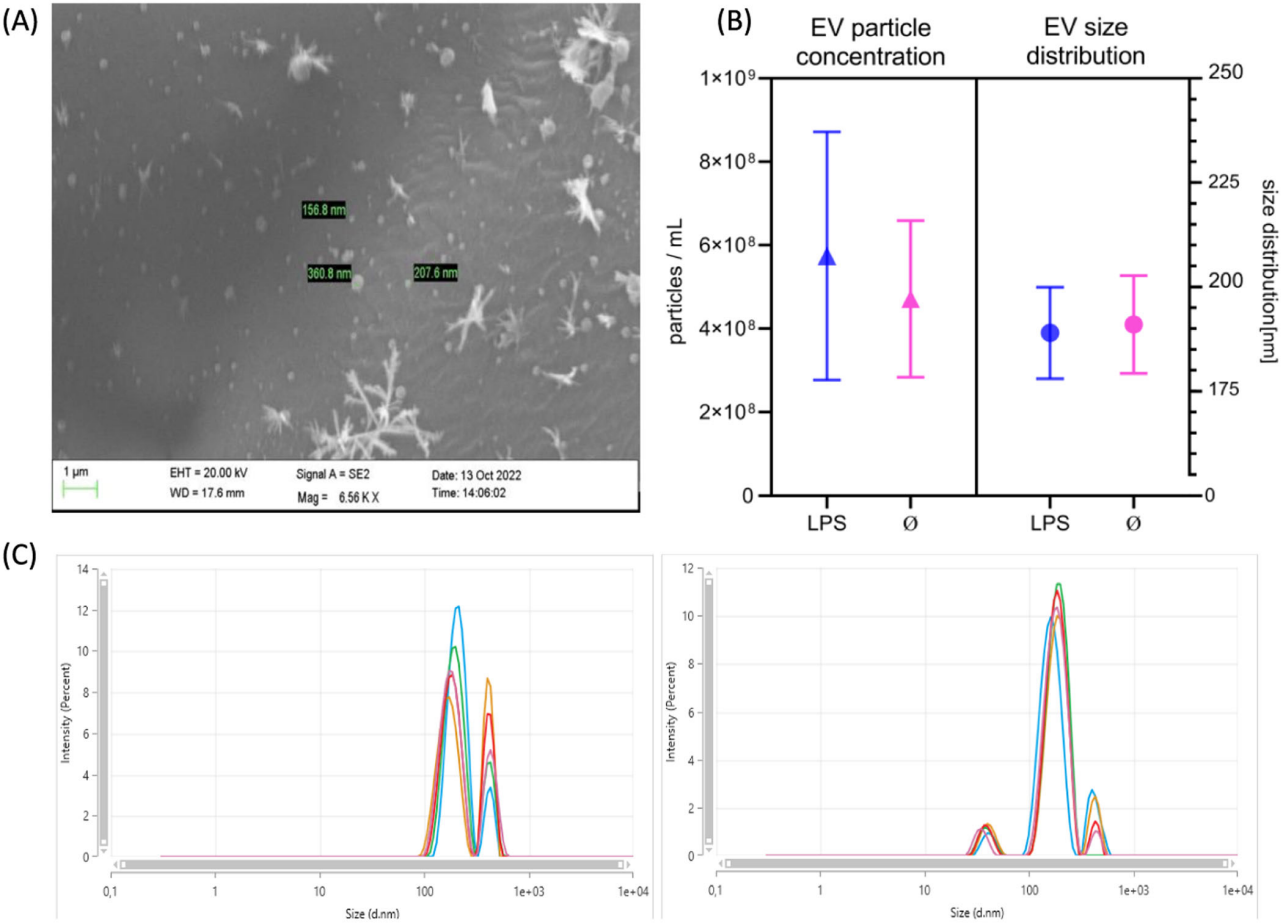
(A) Exemplary SEM picture from purified HGF‐derived EV. (B) Particle concentrations/mL and size distribution of diluted EV preparations. The graphs represent means with 95% CI. (C) Illustration of dynamic light scattering results. Each peak represents a specific size fraction, and the colors represent replicates, indicating reproducible EV purification.

**Figure 2:**
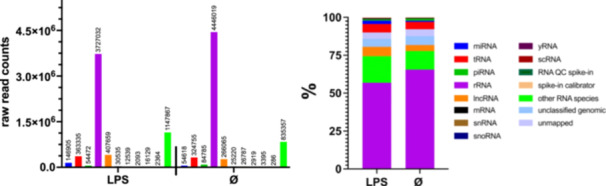
Raw read counts from EV‐derived RNA species.

**Figure 3:**
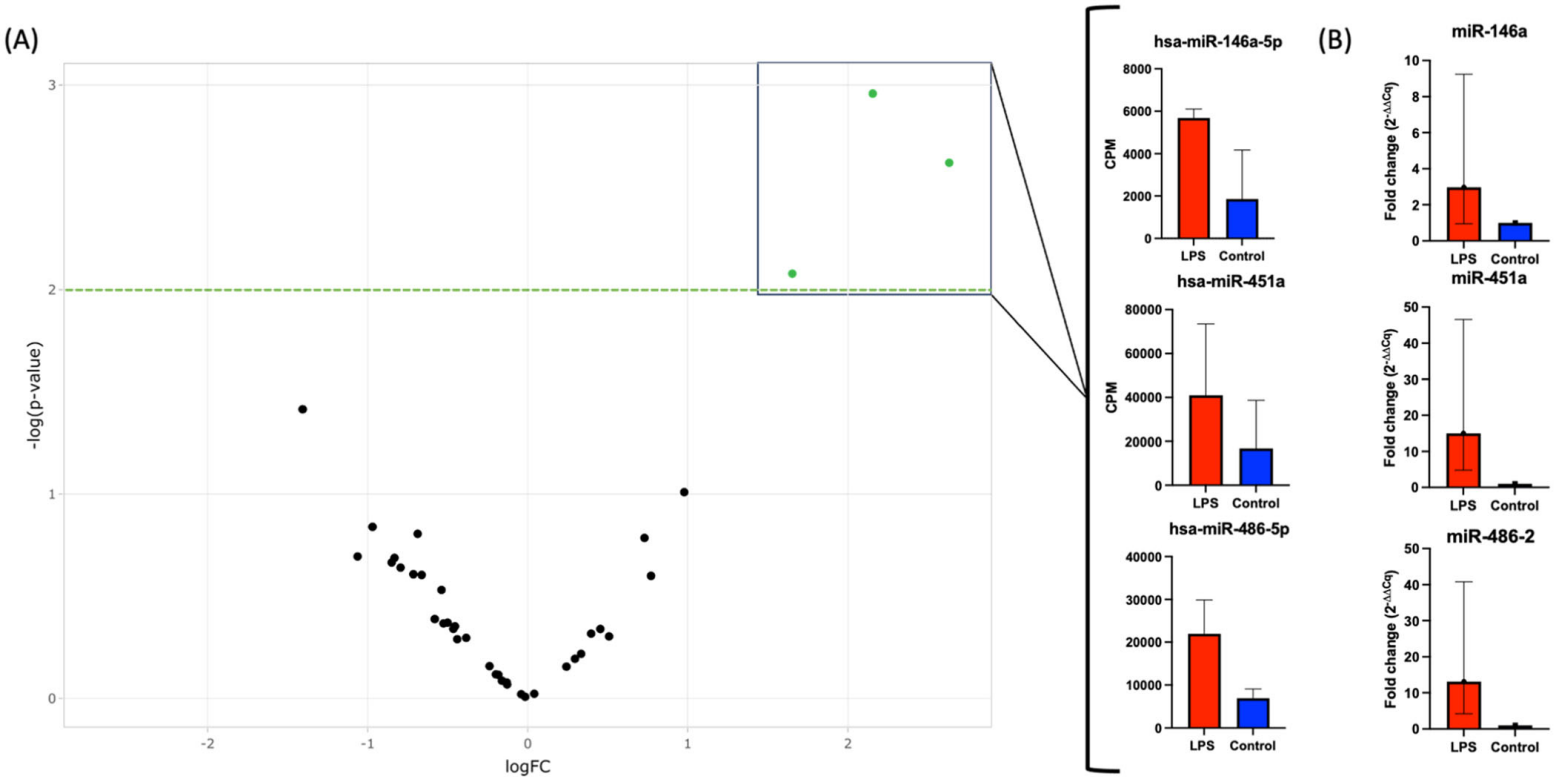
(A) Volcano plot of differentially expressed miRNA. The miRNA with a −log(*p*) above 2 are considered differentially expressed. The graphs show mean counts per million reads (CPM) for each overexpressed miRNA compared to the control group. (B) Verification of miRNA expression in respectively treated HGF cells by RT‐qPCR.

Neither the size (188.98 nm ± 10.97; 191 nm ± 11.72 nm) nor the particle concentration (Control = 4.71 × 10^8^/mL ± 1.88 × 10^8^; LPS = 5.74 × 10^8^/mL ± 2.97 × 10^8^/mL) of EVs differed significantly between LPS‐treated and untreated fibroblasts (Figure 1). The SEM pictures and the DLS analyses show EVs with intact circular vesicular structures in the expected size range between 150 and 400 nm (Figure 1C). A comparison of raw RNA reads yielded an EV‐typical RNA species signature in both groups (Figure 2). Interestingly, a 2.6‐fold increase in miRNA raw read counts was found in the LPS‐derived EVs compared to the unstimulated group. In total, 559 miRNAs were identified, of which 359 were common in both groups. A total of three miRNAs, namely miR‐146a‐5p, miR‐486‐5p, and miR451a, were considered differentially expressed (Figure 3). On the other hand, it is noteworthy that no miRNA were significantly downregulated in the EV derived from LPS‐challenged fibroblasts.

While miR‐146a‐5p was most significantly upregulated in the LPS group, it exhibited the least counts per million reads (∼5000). The other two upregulated miRNA exhibited higher read counts, with ∼20,000 for miR‐486‐5p and around 40,000 for miR‐451a (Figure 3A). These findings were verified by RT‐qPCR, where miR‐146a‐5p was overexpressed 2.5‐fold, miR‐451a was upregulated nearly 14‐fold, and miR‐486‐5p was upregulated 11‐fold in HGF cells treated with bacterial LPS (Figure 3B).

### 3.2 Gene Ontology and KEGG Pathway Enrichment

**Figure 4:**
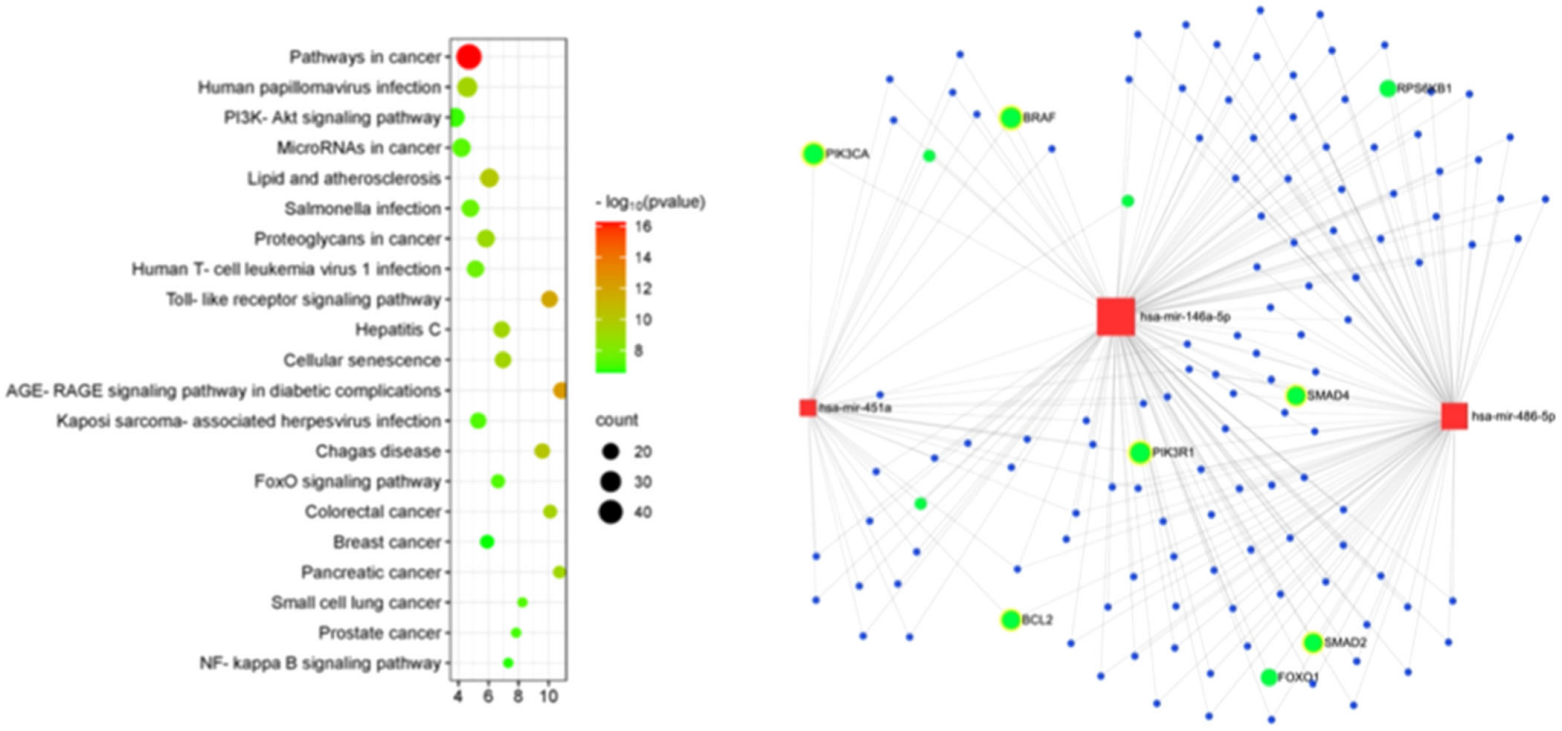
Bubble plot for KEGG pathway enrichment. miRNA‐centric network with validated target genes for miR‐451a, miR‐486 and miR‐146a. Target genes related to pathways in cancer are highlighted in green.

Based on validated targets, a total of 601 target genes were queried for further analyses. The complex interaction relationship between these three miRNAs and their target genes is visualized as a network (Figure 4). miR146a‐5p showed the highest number of overall target genes. Interestingly, enriched KEGG pathway analyses revealed that the targeted genes that are regulated by the EV‐derived DEMs are involved in a variety of pathways related to cancer and inflammatory signaling, for example, toll‐like receptor signaling, PI3K‐Akt signaling or AGE‐RAGE signaling (Figure 4). The cancer‐related genes with predicted interaction are highlighted in Figure 4.

### 3.3 High Expression of miRNAs Derived From LPS‐EVs Is Linked With Reduced Survival in Cancer Patients

**Figure 5:**
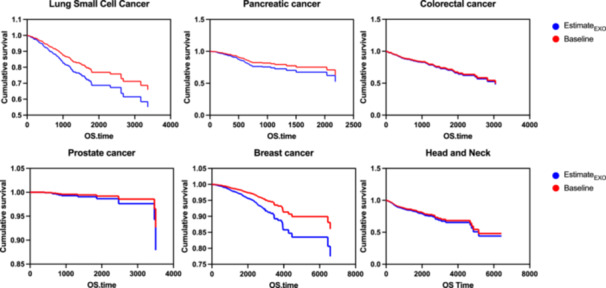
Estimated survival analysis for TCGA cohorts. The baseline (red) survival is plotted against the survival estimate with hazard ratios for miRNA expression applied. OS.time, overall survival time in days.

The differential expression of EV‐derived miRNAs was investigated between normal adjacent and tumor tissues from TCGA cohorts. We retrieved miRNA‐Seq data for six different malignancies associated with our DEM according to target prediction by accessing the GDC data through UCSC Xena (Table 2). Overall, the expression of miR‐146a, miR‐486, and miR451a displayed heterogeneous hazard ratios across different TCGA cohorts. Intriguingly, the estimated survival analysis based on the individual beta‐coefficients of exosome‐derived miRNAs yielded substantially decreased survival rates in the LUAD and BRCA cohorts and slightly decreased outcomes in PRAD and PAAD cohorts. The tumor entities closer to the periodontium, namely HSNC, and COAD, however, exhibited no decreases in survival estimates (Figure 5).

**Table 2:** Hazard ratios and 95% CI for TCGA cohorts according to Cox regression.

	BRCA	COAD	HNSC	PAAD	LUSC	PRAD
hsa‐miR‐146a	1.012 0.8386–1.207	1.087 0.6771–1.771	0.7566 0.6206–0.9228	0.8716 0.6144–1.236	0.6221 0.4215–0.9123	1.102 0.4391–2.577
hsa‐miR‐451a	0.6920 0.4847–1.004	0.8412 0.6252–1.135	0.9479 0.7408–1.224	1.025 0.5126–2.138	1.334 0.8816–2.037	0.9654 0.2199–4.214
hsa‐miR‐486	1.643 1.087–2.411	1.097 0.8166–1.452	1.185 0.8499–1.634	0.5745 0.2289–1.378	0.7956 0.5320–1.181	0.4885 0.1026–2.067
Age at initial diagnosis	1.032 1.018–1.047	1.002 0.9746–1.031	1.021 1.008–1.033	1.024 1.003–1.047	1.014 0.9945–1.034	1.015 0.9233–1.118
TNM stage I	0.4733 0.1067–1.516	1.783 0.6544–6.227	0.7430 0.3168–2.036	n/a	1.003 0.9954–1.009	1.061 1.020–1.127
TNM stage IV	11.29 5.145–25.32	13.88 5.236–47.95	1.409 0.6660–3.643	n/a	1.060 0.8526–1.320	1.339 0.5097–2.228

### 3.4 LPS‐EV From Gingival Fibroblasts Induce Enhanced Proliferation and Epithelial–Mesenchymal Transition in Cancer Cells

**Figure 6:**
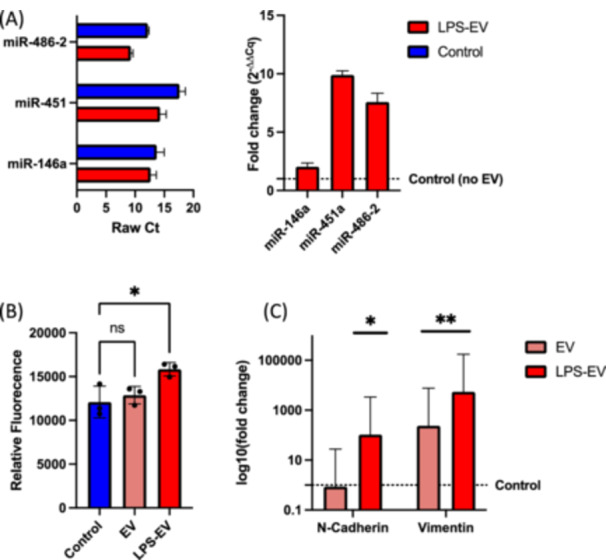
(A) Relative gene expression of the identified miRNA in LnCap cells treated with LPS‐EV or media as measured via RT‐qPCR. The left figure represents raw Ct values, while the right figure presents the relative fold changes compared to the untreated control. (B) Proliferation assay results. (C) Relative expression of N‐Cadherin (*CDH2*) and Vimentin (*VIM*) expression in LnCap cells treated with either media, normal EV or LPS‐EV. *p < 0.005; **p < 0.01.

To verify the in silico analyses, the prostate cancer cell line LnCap was cultured in the presence of LPS‐EV from the abovementioned isolations. Interestingly, the expression of the previously identified miRNA substantially increased between 2‐fold (miR‐146a) and 10‐fold (miR‐451a, Figure 6A). More importantly, the resazurin assay revealed a significant increase of proliferation compared to the control group mediated by the LPS‐EV (*p* = 0.02, Figure 6B). This observation was accompanied by a significant increase in *VIM* and *CDH2* gene expression, two major markers for epithelial‐to‐mesenchymal transition (Figure 6C).

## 4 Discussion

Periodontitis remains a global health burden that significantly influences a plethora of systemic diseases. The molecular mechanisms by which periodontitis exerts negative health consequences remain to be further elucidated. Identifying putative biological processes that bridge this gap will not only further our understanding of the disease but may also lead to the discovery of novel systemic surrogate biomarkers for periodontitis.

In this study, we characterized, for the first time, the EV nucleic acid cargo from gingival fibroblasts under the influence of bacterial endotoxin. The results revealed a specific profile of differentially expressed miRNA, namely miR‐146a, miR‐486, and miR‐451a. miR‐146a and miR‐146b are two evolutionarily conserved genes that belong to the miR‐146 family of miRNAs. Both genes respond to LPS in an NF‐κB‐dependent manner in human monocytes, but only miR‐146a is processed to a mature form (Boldin et al. 2011). For its ubiquitous expression related to inflammatory processes, it is also widely referred to as an “inflamma‐miR.” In immune cells, this miRNA is a posttranscriptional negative signal transduction regulator, mainly targeting interleukin‐1 receptor‐associated kinase 1 within the toll‐like‐receptor pathway (Mortazavi‐Jahromi et al. 2020). Upregulation of miR‐146a has also been witnessed in gingival samples of patients suffering from aggressive periodontitis (Ghotloo et al. 2019). In this regard, significant upregulation of miR‐146a in inflammatory exosomes from gingival fibroblasts aligns with the literature. miR‐486, on the other hand, constitutes a gene with ambivalent functions throughout the organism. By binding to the 3′UTR of its validated targets, like phosphatase and tensin homolog and forkhead transcription factor 1, it has been reported to decrease cardiomyocyte apoptosis and increase kidney cell function (Viñas et al. 2016; Zhu et al. 2019). On the other hand, it is responsible for inhibiting chondrocyte proliferation in osteoarthritis by suppressing the transcriptional regulator Mothers against decapentaplegic homolog 2 (SMAD2). Additionally, miR‐486 binds to and inhibits the CYLD lysine 63 deubiquitinase gene, which is translated into a deubiquitinase responsible for limiting NF‐κB activation via suppression of negative feedback loops (Shi et al. 2018; Song et al. 2013).

Similarly, miR‐451a has been shown to increase the progression of SLE and to be involved in sepsis‐mediated organ injury (Cheng et al. 2017; Geng et al. 2022; Wang et al. 2020). Since most of these disease entities exhibit significant associations with periodontitis (Martínez‐García and Hernández‐Lemus 2021), it is feasible to speculate that exosomes derived from inflamed periodontal tissues may affect the onset and progression of systemic diseases via their respective miRNA cargo. Pharmacokinetic studies further underline this notion, showing that exosomes are bioavailable, stable over long periods, and distributed into every major organ of the body (Manca et al. 2018).

Accounting for nearly 15% of global deaths, cancer represents a major cause of global mortality, and identifying risk factors and contributors to the disease is of vital importance to global health (Lozano et al. 2012). Numerous observational studies have reported a significantly increased cancer risk for periodontitis patients; however, the pathophysiologic foundation of this association remains unclear (Corbella et al. 2018). Intriguingly, the set of miRNAs found in LPS‐derived exosomes in this study was significantly associated with signaling pathways in various forms of cancer. Our estimated survival analysis in disease cohorts from TCGA revealed substantially increased mortality in lung, breast, prostate, and lung cancer but not for colorectal or head and neck cancers. To validate this hypothesis, we cultured cancer cells—a prostate cancer cell line in this case—in the presence of gingival fibroblast LPS‐EV. Interestingly, this treatment led to a substantial overexpression of the previously identified miRNA. Moreover, thusly cultured LnCap cells exhibited increased proliferation and expression of *VIM* and *CDH2*, two genes associated with epitheliam‐mesenchymal transition and thus metastasis (Cheaito et al. 2019; Gravdal et al. 2007). Taken together with our in silico analyses, this indicates that these miRNAs may contribute to the mortality in these cohorts, rendering exosomes transporting the respective miRNAs a putative mechanism by which periodontitis negatively affects malignant diseases. This hypothesis is further emphasized by reports in recent history that miR‐146a constitutes a significant biomarker for triple‐negative breast cancer (TNBC) and may promote the proliferation and invasion of TNBC cells (Chen et al. 2020; Fkih M'hamed et al. 2015). More importantly, breast cancer‐derived exosomes express miR‐146a, which regulates invasiveness and metastasis by targeting cells in the tumor microenvironment (Yang et al. 2020). However, the role of these miRNAs in cancer seems to be conflicting. For example, according to many reports, miR‐451a and miR‐486 are considered tumor suppressors, and their downregulation rather than upregulation seems to be associated with disease progression (Li et al. 2018; Wang et al. 2014).

On the other hand, substantial experimental evidence suggests a major oncogenic role for these miRNAs, especially in lung and prostate cancer, where both inhibit tumor suppressor genes and drive disease progression (Chen et al. 2014; Gao et al. 2018; Yang et al. 2017). Within the entirety of the available observational studies and the results of this investigation, it is reasonable to suggest that exosomes from active periodontal lesions bear all the properties required to exert a negative influence on malignant diseases. Nevertheless, the systemic burden and distribution kinetics of inflammatory oral exosomes remain to be elucidated to support this claim. Unfortunately, identifying and associating serum exosomes with a specific release origin remains a major challenge in contemporary vesicle research that remains to be solved (Li et al. 2020).

Although primary gingival fibroblasts were used for this in vitro study, the results are not easily translatable to clinical settings, where a more heterogeneous composition of periodontal tissues and variable disease progression must be taken into account. Nevertheless, the miRNAs identified in this study have consistently been reported as upregulated in gingival tissues from periodontitis patients, suggesting that the immortalized cell line used may have produced representative results (Lin et al. 2021; Luan et al. 2018; Wang et al. 2019). However, future studies should examine the exosome composition of patients with different forms of periodontitis. For the reasons mentioned above, bias‐free purification of the respective EVs from biological fluid for downstream high‐throughput or functional analyses is not feasible yet.

Validating the size and quantity of exosome preparations, we found that LPS stimulation of fibroblasts did not significantly alter the size distribution or quantity of secreted exosomes. However, we did see a substantial mean increase in particle concentration upon LPS treatment in the supernatant. In a study investigating the effects of inflammation on stem cell‐derived exosome production, Huang et al. (2019) found significantly increased exosome release upon treatment with pro‐inflammatory cytokines. Considering these results, it may be worthwhile to investigate whether inflammation increases EV secretion in periodontal cells. This, however, would require a single‐cell analysis approach to account for the high variability between samples witnessed in this study.

This is the first study to analyze EV cargo in inflamed periodontal fibroblasts. The EV‐associated miRNAs miR‐146a, miR‐451a, and miR‐486 exhibit target genes that may substantially influence a variety of tissues and disease entities. In particular, they seem to significantly affect disease progression in various tumors, indicating a novel mechanism behind the association of periodontitis with cancer. Further validation of these findings in a clinical setting, as well as functional studies with periodontal exosomes in cancer biology, are warranted.

## Author Contributions

D.D., C.L.B., and A.F. conceptualized and designed the study. D.D. and C.L.B. performed the experiments. H.S.B. contributed to data analysis and interpretation. D.P. and P.P.W. provided critical input on methodology and data validation considering sRNA‐Seq and lipid assays. A.F. supervised the project and provided resources. All authors contributed to manuscript drafting and revision and approved the final version for submission.

## Conflicts of Interest

The authors declare no conflicts of interest.

## Data Availability

The data that support the findings of this study are available from the corresponding author upon reasonable request.
